# In‐Plane Overdamping and Out‐Plane Localized Vibration Contribute to Ultralow Lattice Thermal Conductivity of Zintl Phase KCdSb

**DOI:** 10.1002/advs.202402209

**Published:** 2024-07-01

**Authors:** Kai Guo, Juan Zhang, Xiaotong Yu, Yuanxin Jiang, Yang Li, Yuqi Zeng, Ruixiao Lian, Xinxin Yang, Shuankui Li, Jun Luo, Wen Li, Hao Zhang

**Affiliations:** ^1^ School of Physics and Materials Science Guangzhou University Guangzhou 510006 China; ^2^ Key Lab of Si‐based Information Materials & Devices, Integrated Circuits Design Department of Education of Guangdong Province Guangzhou 510006 China; ^3^ School of Information Science and Technology and Department of Optical Science and Engineering and Key Laboratory of Micro and Nano Photonic Structures (MOE) Fudan University Shanghai 200433 China; ^4^ School of Materials Science and Engineering Shanghai University Shanghai 200444 China; ^5^ Interdisciplinary Materials Research Center School of Materials Science and Engineering Tongji University Shanghai 201804 China; ^6^ State Key Laboratory of Photovoltaic Science and Technology Fudan University Shanghai 200433 China

**Keywords:** KCdSb, lattice thermal conductivity, phonon velocity, thermoelectric properties, Zintl phase

## Abstract

Zintl phases typically exhibit low lattice thermal conductivity, which are extensively investigated as promising thermoelectric candidates. While the significance of Zintl anionic frameworks in electronic transport properties is widely recognized, their roles in thermal transport properties have often been overlooked. This study delves into KCdSb as a representative case, where the [CdSb_4/4_]^−^ tetrahedrons not only impact charge transfer but also phonon transport. The phonon velocity and mean free path, are heavily influenced by the bonding distance and strength of the Zintl anions Cd and Sb, considering the three acoustic branches arising from their vibrations. Furthermore, the weakly bound Zintl cation K exhibits localized vibration behaviors, resulting in strong coupling between the high‐lying acoustic branch and the low‐lying optical branch, further impeding phonon diffusion. The calculations reveal that grain boundaries also contribute to the low lattice thermal conductivity of KCdSb through medium‐frequency phonon scattering. These combined factors create a glass‐like thermal transport behavior, which is advantageous for improving the thermoelectric merit of *zT*. Notably, a maximum *zT* of 0.6 is achieved for K_0.84_Na_0.16_CdSb at 712 K. The study offers both intrinsic and extrinsic strategies for developing high‐efficiency thermoelectric Zintl materials with extremely low lattice thermal conductivity.

## Graphical abstract

In this contribution, the significance of the polyanionic framework constructed by [CdSb_4/4_]^−^ tetrahedrons is highlighted in KCdSb on the electronic and thermal transport properties. The couplings of two transverse acoustic modes, as well as high‐lying acoustic branches and low‐lying optical branches slow the phonon diffusion, beneficial for ultralow *κ*_lat_. The thermoelectric properties of KCdSb is first reported, with a maximum *zT* value of 0.6 at 712 K for K_0.84_Na_0.16_CdSb.

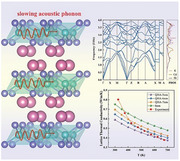

## 1 Introduction

Zintl phases are polar intermetallic compounds named after Eduard Zintl, a German chemist born in 1889.^[^ ^1^ ^]^ This subclass of intermetallics is characterized as valence‐precise semiconductors that involve both ionic and covalent bonding in the lattice. Ionic bonding is related to the electropositive cations (such as alkali, alkali earth, and rare earth metals) that donate electrons to more electronegative elements, which yield extended polyanions or clusters with either covalent or polar covalent bonding, or isolated anions with their full octet.^[^ ^2^, ^3^ ^]^ In the past two decades, Zintl phases have garnered significant attention in the field of thermoelectrics due to their structural and bonding characteristics aligning with the concept of “electron‐crystal, phonon‐glass”.^[^ ^4^, ^5^, ^6^, ^7^ ^]^ Notably, the distinctive components of Zintl phases can simultaneously fulfill different functions within a lattice to realize promising thermoelectric properties, which isevaluated by the dimensionless thermoelectric of merit *zT* = *S^2^σT*/*κ*, where *S*, *σ*, *T*, and *κ* are the Seebeck coefficient, electrical conductivity, absolute temperature, and total thermal conductivity, respectively.^[^ ^8^ ^]^ Correspondingly, efficient thermoelectrics require good electronic transport properties (large power factor *PF* = *S^2^σ*) but low total thermal conductivity *κ* even though they are strongly coupled. The total thermal conductivity *κ* normally consists of the electronic contribution (*κ*_e_) and lattice contribution (*κ*_L_), which stem from carriers and phonons, respectively. In Zintl phases, the polyanionic frameworks primarily governed by covalent interactions are generally regarded as the “electron‐crystal” electronic structure, which plays a crucial role in electronic transport to guarantee moderate carrier mobility and effective mass. This can be observed in compounds such as YbMg_2_Sb_2_,^[^ ^9^, ^10^, ^11^ ^]^ YbZn_2_Sb_2,_^[^ ^12^, ^13^ ^]^ and YbCd_2_Sb_2_,^[^ ^13^, ^14^ ^]^ where the [*B*Sb_4/4_] (*B* = Mg, Zn, and Cd) tetrahedrons mainly contribute to the valence band. Moreover, the bonding strength between *B* and Sb has a vital effect on the carrier mobility and the bandgap width. For thermal transport properties, a popular view is that the diversity of bonding and structural units in Zintl phases often leads to complex structures, resulting in significantly low lattice thermal conductivity *κ*_L_, thereby acting as “phonon‐glass” components. Particularly, in the case of complex Zintl compounds like Yb_14_MnSb_11_, the presence of large unit cells suppresses the contribution of the acoustic branch to thermal conductivity, thus enhancing the thermoelectric figure of merit *zT*.^[^ ^15^, ^16^ ^]^ In addition, the occurrence of local weak bonding can often lead to the interaction between high‐lying acoustic branches and low‐lying optical branches, resulting in a decrease in phonon velocity and thermal conductivity. This phenomenon has been frequently observed in layered and caged‐like Zintl phases, such as Mg_3_Sb_2_ and BaCu_2_X_2_ (X = Se, Te).^[^ ^17^, ^18^, ^19^ ^]^

**Figure 1:**
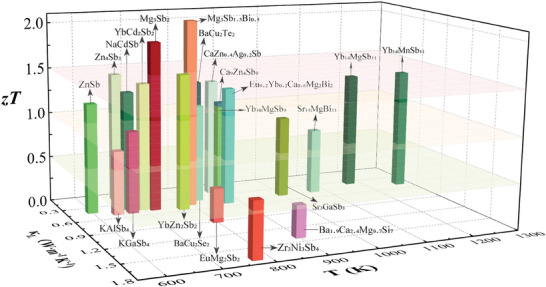
The peak *zT* values for state‐of‐art Zintl thermoelectrics. The greenish color and reddish color represent p‐type and n‐type thermoelectric materials, respectively.^[^ ^9^, ^10^, ^11^, ^12^, ^13^, ^14^, ^15^, ^16^, ^17^, ^18^, ^19^, ^20^, ^21^, ^22^, ^23^, ^24^, ^25^, ^26^, ^27^, ^28^, ^29^, ^30^, ^31^, ^32^, ^33^, ^34^, ^38^, ^39^, ^40^, ^41^, ^42^, ^43^ ^]^

So far, numerous Zintl phases have been extensively studied for their thermoelectric properties, with peak *zT* values >1. Representative phases include the 1‐2‐2,^[^ ^9^, ^10^, ^11^, ^12^, ^13^, ^14^, ^18^, ^19^, ^20^, ^21^, ^22^, ^23^ ^]^ 14‐1‐11,^[^ ^15^, ^16^, ^24^, ^25^, ^26^ ^]^ 10‐1‐9,^[^ ^27^ ^]^ 9‐4‐9,^[^ ^28^ ^]^ and 1‐1‐1 types,^[^ ^29^, ^30^, ^31^, ^32^, ^33^ ^]^ as depicted in **Figure** **1**. Particularly, the n‐type Mg_3_(Sb, Bi)_2_ Zintls has recently been reported with a record‐high average *zT* > 1.5 (300–798 K) and a maximum *zT* of 2.04 at 798 K, attributing to the modulation of charge carriers and phonon transport through the introduction of Nb/Ta inclusions at grain boundaries.^[^ ^34^ ^]^ This demonstrates the huge potential of thermoelectric applications for state‐of‐the‐art Zintl materials. Overall, the thermoelectric potential of Zintl phases can be ascribed to the moderate electronic transport properties and low lattice thermal conductivity *κ*_L_ (<0.6 W (m K)^−1^). Lattice thermal conductivity, *κ*_L_, can be expressed using the analogy of the kinetic theory of gases as,^[^ ^35^ ^]^

(1) $$\;\kappa_{l}=\frac{1}{3\Omega }\;\sum_{\lambda q}C_{\lambda q}v_{\lambda q}^{2}\tau_{\lambda q}$$

where Ω is the crystal volume, λ is the phonon mode, *C*_λ*q*_,*v*_λ*q*_,τ_λ*q*_ are the heat capacity, the group velocity, the phonon lifetime for each phonon mode, respectively. Therefore, inherently low lattice thermal conductivity *κ*_L_ stems from small heat capacity (*C*_v_), slow phonon velocity (*v*), or short phonon relaxation times (*τ*). In typical thermoelectric materials with a Debye temperature ranging from 100–200 K, the heat capacity approaches 3*R*/*M* above room temperature according to the Dulong‐Petit law (*R* represents the universal gas constant, and *M* is the molecular mass). As a result, the theoretical value of 3*R*/*M* is often used to calculate *κ*_L_, highlighting the importance of heavier components with large molecular mass. An interesting exception is observed in fast‐ion conductors like Cu_2_Se, where partial acoustic transversal phonon modes are lost, leading to an ultralow heat capacity closer to 2*R*/*M*.^[^ ^36^ ^]^ When it comes to phonon velocity and phonon relaxation time, weak interactions, heavy components, large anharmonicity, and multi‐scale defect scattering are preferred for achieving low *κ*_L_. Our previous work focusing on 1‐2‐2 type Zintl phases reveals the impact of mean atomic mass, crystal structure, and bonding anisotropy on the lattice thermal conductivity *κ*_L_.^[^ ^37^ ^]^ However, the expression of low *κ*_L_ in Zintl phases remains vague, especially for Zintl phases like Mg_3_Sb_2_ and NaCdSb with light compositions and simple structures, which go against the regular paradigms.^[^ ^17^, ^31^ ^]^

In this study, we emphasize the effects of the anionic group on phonon transport. Specifically, we take the 1‐1‐1 type Zintl phase KCdSb as an example and systematically investigate its lattice thermal conductivity using a combined theoretical and experimental approach. We highlight the critical roles played by [CdSb_4/4_]^−^ tetrahedrons, which mainly contribute to the acoustic branch vibrations. The coupling between two transverse wave acoustic branches slows the phonon diffusion, exhibiting phonon overdamping behaviors. Meanwhile, low‐lying optical branches resulting from the vibration of K coupled with high‐lying acoustic branches, further reduce the phonon velocities and ultimately affect the thermal transport properties.

## 2 Results and Discussion

**Figure 2:**
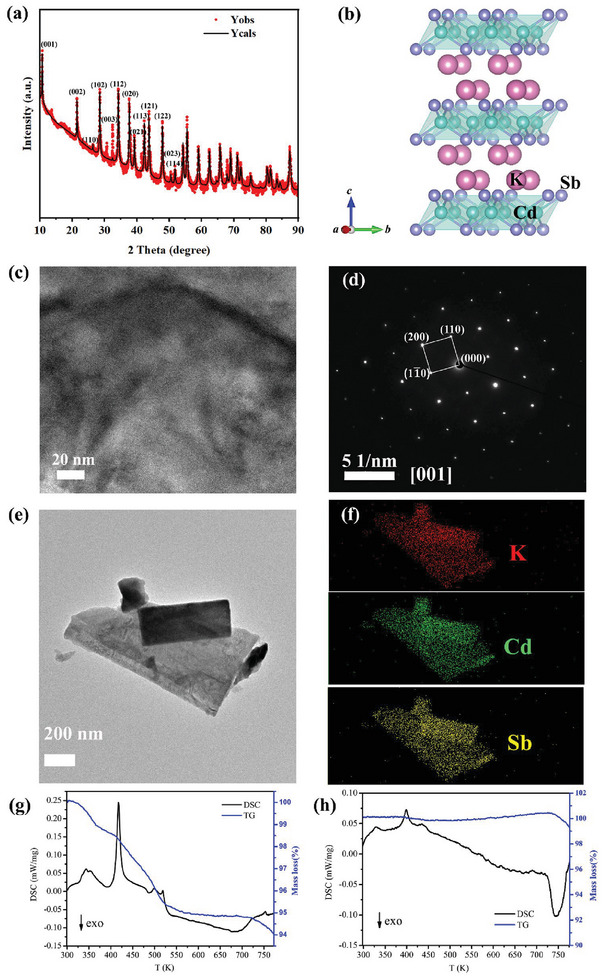
a) The powderXRD patterns for the as‐synthesized KCdSb sample. b) The crystal structure of KCdSb. c) TEM image and d) selected area electron diffraction pattern of KCdSb. e) A typical crystal grain of as‐synthesized KCdSb with a diameter of several hundred nanometers. f) The corresponding elemental mappings e) for K, Cd, and Sb. The thermogravimetric analysis and differential scanning calorimetry (TG‐DSC) data were collected in the temperature range of 300–770 K for the samples weighed in the air (g) and under Ar atmosphere (h), respectively.

Narrow‐bandgap semiconductors with low lattice thermal conductivities have long captured the interest of thermoelectric researchers since the mid‐20th century. Prominent examples include Bi_2_Te_3_,^[^ ^44^ ^]^ PbTe,^[^ ^8^ ^]^ skutterudite,^[^ ^45^ ^]^ argyrodite,^[^ ^46^, ^47^ ^]^ and Zintl phases.^[^ ^37^ ^]^ Zintl phases are considered to be valence‐precise semiconductors with the necessary small bandgaps for thermoelectric considerations, where polyanionic frameworks function as electronic structures. The covalent or polar covalent bonding within these polyanionic frameworks can reach a compromise between carrier mobility and effective mass. However, the significant influence of polyanionic frameworks on thermal transport properties has been overlooked for a considerable period of time. Here, we successfully synthesized the CdSb‐based Zintl phase compound KCdSb through elemental reaction (**Figure** **2**a). In contrast to LiCdSb and NaCdSb, KCdSb adopts a tetragonal structure with the space group of *P*4/*nmm*, as shown in Figure 2b. All major diffraction peaks in the X‐ray diffraction (XRD) patterns are well‐indexed, with the exception of a weak peak ≈30 degrees, which is likely attributed to KOH from hydrolysis. Rietveld refinements were conducted, revealing weak grain orientation with the calculated lattice parameters of *a* = 4.7777(1) Å, *c* = 8.2773(3) Å (Figure S1, Supporting Information). The preferred orientation has negligible influence on the transport properties since the insignificant texture is observed, similar to other Zintl systems (Figure S2, Supporting Information).^[^ ^14^, ^19^ ^]^ Transmission electron microscopy (TEM) measurements also confirm the successful synthesis of KCdSb (Figure 2c,d). The lattice parameters deduced from the selected electron diffraction are well agreed with those calculated from XRD data. Furthermore, homogeneous element distribution for a typical crystal grain can be identified, as can be seen in Figure 2e,f. Some area shows elemental aggregation due to the stacking of two grains.

The resistance of the KCdSb sample to air was evaluated using TG‐DSC measurements, where two samples were weighed in air (Figure 2g) and in a glovebox (Figure 2h), respectively. Multistep mass losses are identified below 720 K for the sample exposed to air for an extended period, while the mass remained constant until 720 K for the sample preserved in an isolated air environment. The mass losses below 370 K (≈1.2%) and between 370–550 K (≈3.8%) in Figure 2g corresponding to the endothermic peaks are attributed to the evaporation of adsorbed water, and decomposition of potassium hydroxide. Therefore, it can be concluded that KCdSb powders readily react with water in the air and should be carefully stored in a glovebox filled with N_2_ or Ar. Furthermore, both samples showed a tendency to lose their masses above 720 K, evidencing the decomposition of KCdSb. It points out the maximum measured temperature of 720 K in the following electronic and thermal transport properties.

**Figure 3:**
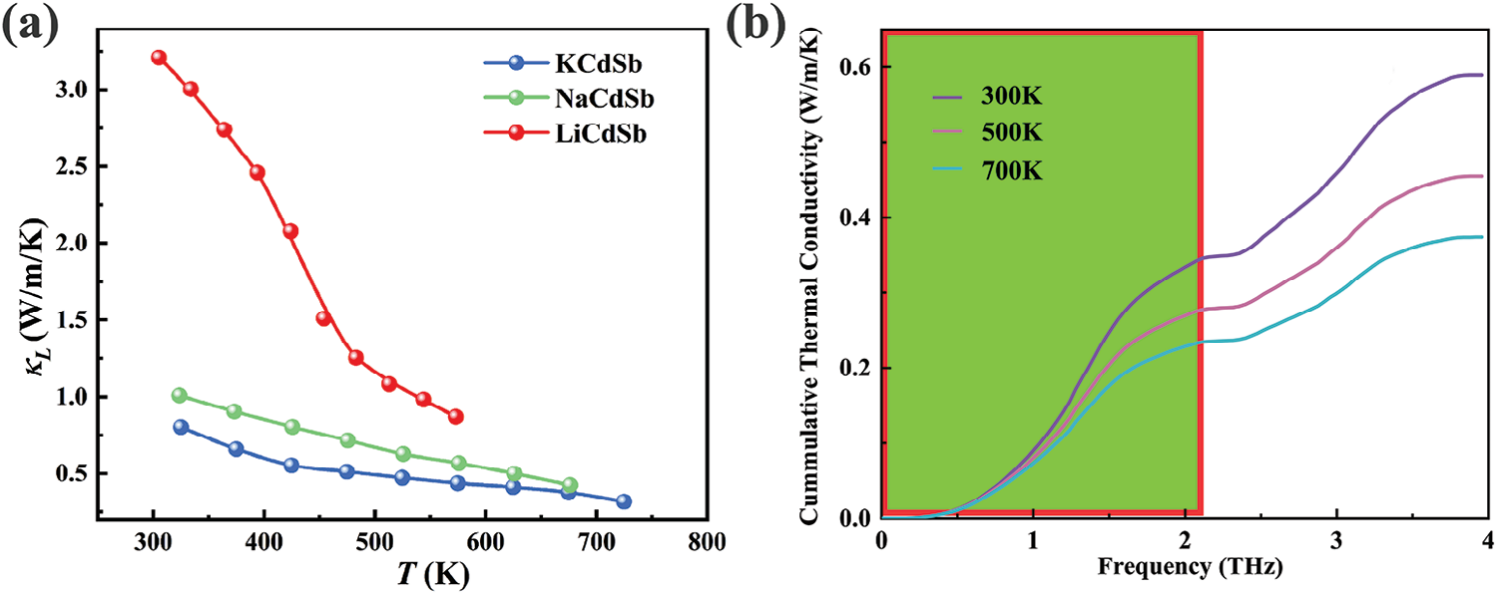
a) The lattice thermal conductivity *κ*_L_ of *A*CdSb (*A* = Li, Na, K) as function of temperature. b) cumulative lattice thermal conductivity of KCdSb.

Interestingly, all three CdSb‐based compounds ACdSb (A = Li, Na, K) exhibit remarkably low lattice thermal conductivity in simple but distinctive structures, with values below 3 W (m K)^−1^ at 323 K and 1 W (m K)^−1^ at 573 K (**Figure** **3**a). As the atomic number of the Zintl cations increases, the lattice thermal conductivity (*κ*_L_) progressively decreases. This trend is especially pronounced in the case of KCdSb, with *κ*_L_ approaching the limit observed in glasses at high temperatures (*κ*_L‐Cahil_ = 0.3 W (m K)^−1^). The primary reason for this behavior is attributed to the low phonon velocity, which approximates the speed of sound in solids. The average speeds of sound for the three compounds are quite similar and relatively low, comparable to those of well‐known thermoelectric materials like PbTe (**Table** **1**),^[^ ^48^ ^]^ which are consistent with the theoretically calculated sound velocity of KCdSb, *v*_l_ = 3432 m s^−1^, *v*_t_ = 1934 m s^−1^, *v*_s_ = 2152 m s^−1^. In the case of LiCdSb, the large atomic displacement parameters of Li^+^ ions are responsible for a “liquid‐like” phonon transport, leading to the low *κ*_L_ in a highly symmetric cubic lattice. On the other hand, for NaCdSb, the fluctuation of the bonding length and strength between Cd and Sb atoms significantly slows down phonon diffusion and suppresses *κ*_L_. However, the origins of the glass‐like thermal conductivity observed in KCdSb still need to be further investigated, which favors exploration of thermal barrier coating and high‐performance thermoelectric materials.

**Table 1:** The measured longitudinal‐wave speed of sound and traverse‐wave speed of sound for NaCdSb and KCdSb. ^*^ stands for the calculated values from the phonon spectrum.

Composition	Longitudinal‐wave speed of sound [*v*_l_, m s^−1^]	Traverse‐ wave speed of sound [*v*_t_, m s^−1^]	Average speed of sound [*v*_s_, m s^−1^]	Cd‐Sb bonding distance [Å]	Volume per atom [Å^3^]
LiCdSb	3345^*^	1469^*^	1658^*^	2.846	23.65
NaCdSb	2836	1564	1743	2.876–2.968	26.63
KCdSb	2688	1565	1736	2.857	31.42

Herein, the method of combining experiments with theory was utilized to shed light on the origins of low *κ*_L_ in Zintl phase KCdSb. To precisely calculate the inter‐atomic force constants (IFCs) at finite temperatures for materials, the temperature‐induced effects should be considered, which generally not only determine the phonon population described by the Bose‐Einstein statistics but also may induce changes in crystal volumes and intrinsic anharmonic phonon–phonon interactions. Here the temperature‐dependent IFCs for KCdSb were calculated by the temperature‐dependent effective potential (TDEP) method,^[^ ^49^, ^50^ ^]^ which fits the IFCs by a least‐square algorithm from the force‐displacement datasets calculated by the ab initio packages in a thermostatic supercell. To account for the temperature‐induced volumetric expansions, the quasi‐harmonic approximation (QHA) method was used to correct the crystal volumes of KCdSb at finite temperatures.^[^ ^51^ ^]^ As a result, the renormalized IFCs and phonon dispersion for Zintl‐phase KCdSb at room temperature considering temperature‐induced changes in anharmonicity and crystal volumes were obtained. Figure 3b presents the cumulative lattice thermal conductivity as a function of frequency. It is evident that this value increases rapidly as the frequency rises, until reaching a plateau between 2 and 2.4 THz. The cumulative lattice thermal conductivity saturates when the frequency exceeds 4 THz, corresponding to 0.65, 0.49, and 0.40 W (m K)^−1^ at 300, 500, and 700 K, respectively, which are very close to the experimental values in Figure 3a. The former (0–2.0 THz) results from the contribution of the acoustic phonon modes while the latter (2.4–4.0 THz) mainly originates from the the optical phonon modes. As the temperature increases, the cumulative lattice thermal conductivity decreases due to the softening of phonon modes and stronger anharmonic interactions. At 300, 500, and 700 K, the acoustic phonon modes with frequencies <2.0 THz contribute 56.5%, 58.6%, and 61.7%, respectively, to the total thermal conductivity (*κ*_L_). Therefore, one can conclude that acoustic phonon modes play a major role in determining the lattice thermal conductivity while optical phonon modes are also important for phonon transport.

**Figure 4:**
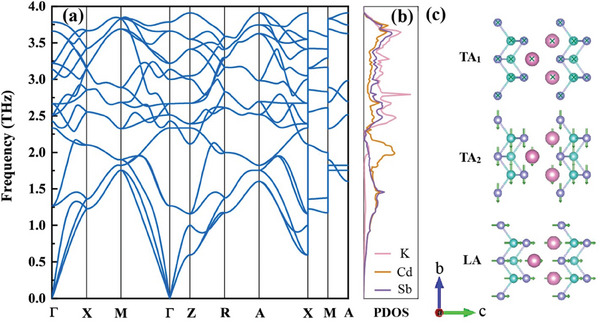
a) The phonon dispersions and b) phonon density of states of KCdSb at room temperature. c) Atomic displacement of three acoustic branches.

**Figure** **4**a,b presents the phonon spectra and phonon density of states for KCdSb at room temperature. Notably, the phonon dispersion shows no imaginary frequencies, indicating the thermal stability of KCdSb at room temperature. As shown in Figure 4b, the low‐lying (<2.3 THz) atomic vibrations are predominantly governed by the quasi‐three‐sublayer [CdSb_4/4_]^−^ tetrahedron structures. These structures contribute to the three acoustic branches, including two transverse acoustic branches (TA) and one longitudinal acoustic branch (LA) vibrating perpendicularly to the sublayer plane (Figure 4c). Similar behaviors have also been observed in LiCdSb and NaCdSb, where the polyanionic group Cd‐Sb is responsible for the acoustic branches.^[^ ^31^, ^32^ ^]^ Furthermore, a coupling between the two TA modes can be identified, which results in not only a reduction in the diffusion velocity of acoustic phonons but also in‐plane overdamping behaviors. Thus, the acoustic phonons contribute only 0.23 W (m K)^−1^ at 700 K to the cumulative thermal conductivity. Additionally, the K^+^ ions distributed between the [CdSb_4/4_]^−^ tetrahedron sublayers play a significant role in the high‐lying region. KCdSb has the largest volume per atom, indicating weak binding of K to the Cd‐Sb framework (Table 1). Interestingly, the vibration of K^+^ ions in this compound shows strong frequency dependence, similar to KCu_5_Se_3_.^[^ ^52^ ^]^ This out‐plane localized vibration results in a coupling between the high‐lying acoustic branch and low‐lying optical branch, impeding the phonon diffusion between the K cations and Cd‐Sb groups and therefore contributing to thermal resistivity. Overall, the coupling between the acoustic and optical branches, as well as the interaction between the transverse and longitudinal acoustic branches, have been identified, leading to decreased phonon velocity and low *κ*_L_.

**Figure 5:**
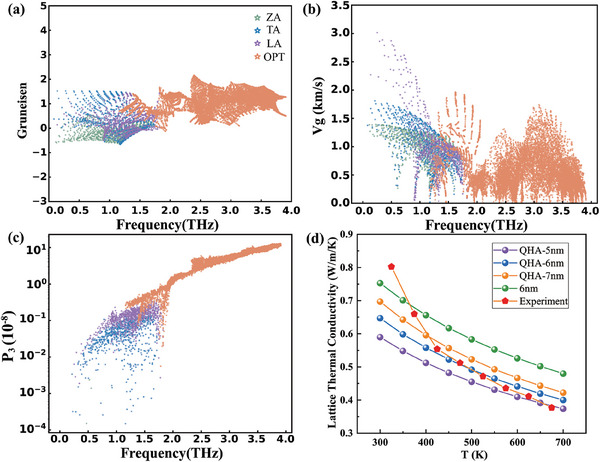
The calculated a) mode Gruneisen parameters γ, b) phonon group velocity, and c) total phase space *P*_3_ for three‐phonon processes of Zintl‐phase KCdSb at room temperature. d) The calculated temperature‐dependent lattice thermal conductivity of KCdSb.

As the above‐mentioned formula, based on the kinetic theory, the lattice thermal conductivity strongly depends on the heat capacity, group velocity, and phonon lifetime of each phonon mode. The mode‐ and momentum‐resolved phonon group velocity *v*_λ*q*_ can be calculated from the phonon dispersion, which is obtained from the harmonic IFCs. Generally, the lattice thermal conductivity *κ*_L_ in semiconductors without significantly large acoutic‐optic phonon bandgap is dominantly determined by the three‐phonon (3 ph) interactions, and then the third‐order IFCs obtained by the TDEP method can be used to calculate the mode‐ and momentum‐resolved phonon lifetime $\tau_{\lambda q}^{3ph}$. To reveal the underlying mechanisms of 3ph‐interaction‐limited *κ*_L_, the mode‐ and frequency‐dependent Grüneisen parameters γ, phonon group velocity and total phase space for 3 ph processes of KCdSb were calculated and shown in **Figure** **5**. As shown in Figure 5a, the Grüneisen parameters for acoustic phonon modes are relatively smaller than those for optical phonon modes, representing their weaker anharmonic interactions. However, as shown in Figure 5b, the phonon velocities for acoustic phonon modes are overally larger than those for optical phonon modes, especially for the longitudinal acoustic (LA) phonon modes. As for the total phase space for 3 ph processes *P*_3_, the optical phonon modes possess more scattering channels than acoustic phonon modes. Therefore, it is reasonable to conclude that the contribution from low‐lying acoustic phonon modes dominate the *κ*_L_ in KCdSb, Therefore, it is reasonable to conclude that the contribution from low‐lying acoustic phonon modes, arising from the vibration of [CdSb_4/4_]^−^ tetrahedron frameworks, dominate the *κ*_L_ of KCdSb.

Furthermore, for the KCdSb samples fabricated here are polycrystalline, and thus the grain‐boundary scattering may play an important role in *κ*_L_. Here a simple grain‐boundary scattering treatment was adopted, in which the phonon lifetime limited by the grain‐boundary scattering τ^*B*^, was approximated by τ^*B*^ = *v_g_* /*L*, with *L* the boundary mean‐free path (MFP). The total phonon lifetime τ_λ ***q***_ can be obtained following the Matthiessen's rule, i.e.,^[^ ^53^ ^]^

(2) $$1/\tau_{\lambda q}\approx 1/\tau_{\lambda q}^{3ph}+\frac{1}{\tau^{B}}$$

The calculated temperature‐dependent lattice thermal conductivity *κ*_L_ as a comparison to the experimental results are shown in Figure 5d. For comparison, *κ*_L_ was calculated with and without considerations of volume‐expansion effects and with the boundary MFP of 5, 6, and 7 nm, and it demonstrates in Figure 5 that, the volume‐expansion effects described by the QHA method reduce *κ*_L_ due to the weaker IFCs when temperature increases, while large MFP enhances *κ*_L_ due to fewer scattering events. Since the calculated Debye temperatures Θ for KCdSb is 201 K, the *κ*_L_ generally demonstrates *T* ^−1^ behavior above Θ due to the dominant contribution from Umklapp 3 ph scattering processes to the thermal conductivities. The experimental lattice thermal conductivity of 0.82 W (m K)^−1^ at 300 K shows a larger derivation from our calculated results. However, our measured data are in good agreement with the case of QHA+6 nm at temperatures ranging from 400 to 670 K by simultaneously considering the volume‐expansion effect and the phonon average MFP of 6 nm, demonstrating the important role of grain‐boundary scattering. Additionally, *κ*_L_ manifests the relation of *T* ^−0.60^, which has probably resulted from the corrections of changes of crystal volumes and the strong grain‐boundary scattering.

**Figure 6:**
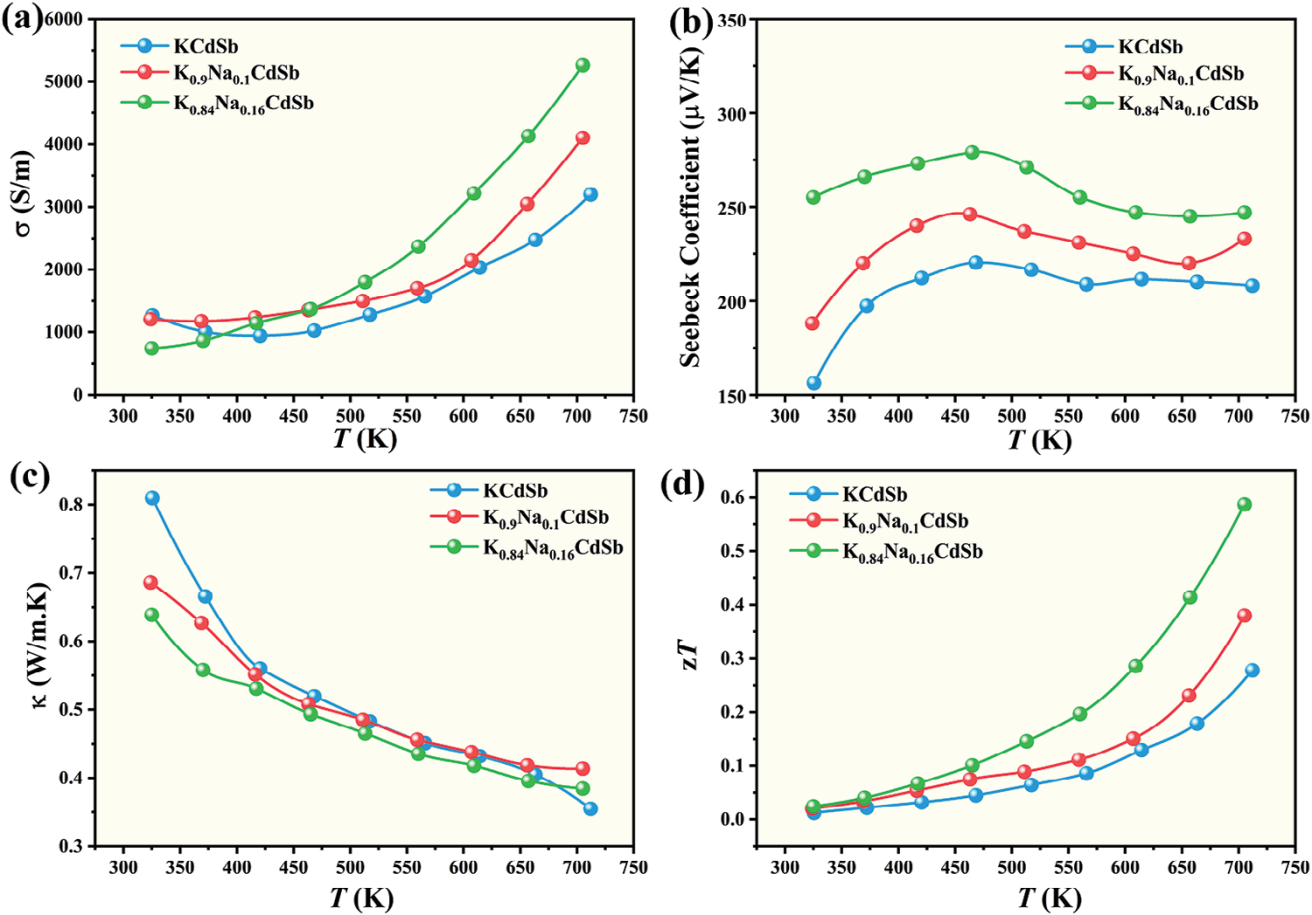
The temperature‐dependent a) electrical conductivity, b) Seebeck coefficient, c) total thermal conductivity, d) figure of merit *zT* for pristine and Na‐alloyed KCdSb.

**Figure 7:**
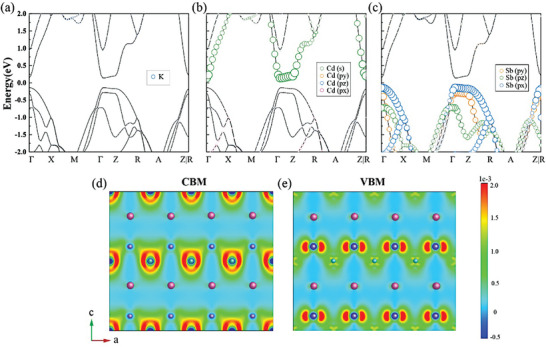
The individual contributions of a) K, b) Cd, and c) Sb to the energy band of KCdSb, d,e) electron density distribution of CBM and VBM.

**Figure** **6** illustrates the temperature‐dependent electrical conductivity, Seebeck coefficient, thermal conductivity, and figure of merit *zT* of pristine and Na‐alloyed KCdSb. The electrical conductivity (*σ*) of KCdSb decreases from 1814 S m^−1^ at 300 K to 942 S m^−1^ at 420 K, before increasing to 3197 S m^−1^ at 712 K. This transition is probably attributed to the intrinsic excitation. Na alloying enhances the electrical conductivity, particularly at high temperatures, due to the increase of hole concentration (Figure S3, Supporting Information). The positive Seebeck coefficients within the measured temperature range indicate that the hole dominates the electronic transport, confirming KCdSb as a p‐type semiconductor. In contrast to *σ*, the Seebeck coefficient (*S*) tends to decrease above 400 K due to increased hole concentration in pristine KCdSb. Interestingly, the substitution of Na significantly enhances *S*, likely due to multiband behavior. **Figure** **7** shows that the valence band maximum (VBM) is dominated by the more dispersive *p*_x_ orbital of Sb, while the conduction band minimum (CBM) is predominantly attributed from the *s* orbital of Cd, highlighting the important role of Cd‐Sb framework in the in‐plane charge transport. Additionally, the *p*_y_ and *p*_z_ orbitals of Sb may contribute to electronic transport when the Fermi level shifts toward lower energies, a manipulation achievable through carrier concentration. Consequently, Na alloying triggers the lighter band *p*_y_ and *p*_z_ orbitals of Sb, beneficial for increasing hole effective mass. Our calculations indicate that the charge densities for both n‐ and p‐type KCdSb tend to mainly distribute within the in‐plane direction formed by Cd‐Sb framework, and those distributions along the out‐of‐plane direction are weak, thus the interlayer coupling between adjacent Cd‐Sb frameworks indeed via K sublayer is less pronounced. The charge‐density differences along in‐plane and out‐of‐plane directions result in high anisotropies in both electronic and thermal transport properties of KCdSb, and much lower carrier mobilities and thermal conductivities along the interlayer direction can be expected (Figure S4, Supporting Information). Thus, texture engineering can be a potential route to optimize the thermoelectric properties of polycrystalline KCdSb.

The power factors, derived from electrical conductivity and Seebeck coefficient, are substantially lower than those of traditional Zintl compounds such as EuZn_2_Sb_2_ and YbCd_2_Sb_2_.^[^ ^6^ ^]^ This can be attributed to the relatively low electrical conductivity of KCdSb. When combined with its ultra‐low thermal conductivity, the figure of merit zT reaches 0.27 at 712 K for pristine KCdSb. The introduction of Na significantly enhances the thermoelectric properties, resulting in a peak *zT* of 0.6 for K_0.84_Na_0.16_CdSb at the same temperature. Our research offers a straightforward approach to developing high‐performance Zintl thermoelectric materials with low lattice thermal conductivity, achieved by leveraging weak and variable interactions within the polyanionic framework alongside heavy and weakly bound Zintl cations.

## 3 Conclusion

In this work, our main objective is to provide thorough insights into the various factors that contribute to the ultralow lattice thermal conductivity observed in Zintl phase KCdSb. We have accomplished this by utilizing a combination of theoretical calculations and experimental characterizations. Similar to LiCdSb and NaCdSb, the polyanionic framework Cd‐Sb plays a crucial role in determining the electronic and thermal transport properties of KCdSb. We have discovered that the coupling of two transverse acoustic (TA) modes facilitates the reduction of acoustic phonon diffusion, which corresponds to the atomic vibrations along the quasi‐three‐sublayer [CdSb_4/4_]^−^ tetrahedron structures. Moreover, the K^+^ ions exhibit a notable frequency dependence in the phonon spectra, leading to the coupling between high‐lying acoustic branches and low‐lying optical branches. The low phonon velocities of both the acoustic and optical phonons contribute to the intrinsic low lattice thermal conductivity of KCdSb. Furthermore, our calculations indicate the significant role of grain boundaries in scattering medium‐frequency phonons and reducing phonon lifetime, ultimately leading to a further suppression of lattice thermal conductivity. As a result of these combined factors, KCdSb demonstrates glass‐like thermal transport behaviors, which are highly advantageous for achieving a large thermoelectric figure of merit, *zT*. Notably, Na‐alloyed KCdSb has achieved a maximum *zT* value of 0.6 at 712 K, showcasing its immense thermoelectric potential. This body of work represents both intrinsic and extrinsic approaches toward obtaining low thermal conductivity, which can greatly benefit the exploration of thermal barrier coatings and thermoelectric materials.

## 4 Experimental Section

### Sample Synthesis

The high‐purity metals K (99.9%), Na (99.9%), Cd (99.9999%), and Sb (99.99999%) were weighed according to K_1‐_ *_x_* Na*_x_* CdSb (*x* = 0, 0.10, 0.16) in the glove box filled with high‐purity argon (Mbraun‐UNIlab Pro, H_2_O < 0.1 ppm, O_2_ < 0.1 ppm). A slight excess of K was utilized as compensation for the loss of potassium during the reaction. These raw materials were put in a BN crucible, which was then transferred into a sealed quartz tube. These assemblies were placed into a muffle furnace (KSL‐1200X Hefei Kejing Material Technology Co., Ltd) for chemical reaction. The sample was first heated to 373 K at a heating rate of 1 K min^−1^, and then kept at this temperature for 60 min to promote the uniform mixture of various elements under the critical temperature of the melting point of potassium. Subsequently, the temperature was increased to 973 K at a heating rate of 1 K min^−1^ and held for three days. Eventually, the sample was cooled to room temperature with a slow cooling rate of 1 K min^−1^. To avoid oxidation and hydrolysis, the resultant powders were ground in the glove box for further hot‐pressing sintering with a maximum temperature of 673 K and a maximum pressure of 70 MPa by using graphite dies (*Φ* = 10 mm).

### Characterization

Powder XRD data were collected using Bruker D8 Advance to examine the phase composition and structure (Cu target, *λ* = 1.5418 Å) in a vacuum. The measured 2*θ* range was from 9° to 90° with a step of 0.02°. The structure refinements were carried out by the Rietveld method with the software of GSAS‐II.^[^ ^54^ ^]^ The stability of KCdSb was examined by a synchronous thermal analyzer (STA 449F3, Netzsch) from room temperature to 750 K. One sample was weighed in air, and another sample was weighed in the glovebox to evaluate the ability of the hydrolyzing resistance. The microstructure, corresponding electron diffraction, and element mapping were characterized by transmission electron microscopy (TEM, JEM‐F200, JEOL) that was operated at an accelerating voltage of 200 kV and equipped with two silicon drift detectors (SDD). The Seebeck coefficient and electrical conductivity were simultaneously measured by ZEM‐3 (ULVACRIKO) in the temperature range of 323–673 K. Thermal diffusivity *λ* measurements were conducted by a laser pulse conductivity apparatus (LFA467, Netzsch), which was utilized to calculate the total thermal conductivity according to the formula *κ* = *λ C*p *d*, where *C*p is the heat capacity estimated from the Dulong‐Petit law and *d* is the density achieved from the Archimedes method with alcohol as the immersed liquid. The sound velocity was obtained using an ultrasonic echometer (UMS‐100, TECLAB, France) at room temperature. The Hall coefficients at room temperature were collected in an applied magnetic field of 0.8 T with Linseis Sorensen dlm40–75e.

### Calculations

The TDEP technique was utilized to determine the IFCs at finite temperatures, in which both the harmonic and anharmonic force constants were fitted as functions of temperature, and the supercell was set as 3 × 3 × 2. Then, the obtained 2nd and 3rd force constants at 300 K were used to calculate lattice thermal conductivity. The QHA method was performed on a grid comprising temperatures ranging from 300 to 800 K to determine the temperature‐dependent volume. In QHA model, the Helmholtz free energy (F) was calculated by,

(3) $$F\;\left(V,T\right)=E_{stat}\;\left(V\right)+F_{el}\left(V,T\right)+F_{vib}\left(V,T\right)$$

*F*(*V*, *T*) is the Helmholtz free energy, *E_stat_* (*V*) represents the total energy of the system at its ground state for a given volume, *F_el_* (*V*,*T*) and *F_vib_* (*V*,*T*) denote the electronic and lattice ion vibrational contributions to the free energy, respectively. The Gibbs free energy under constant pressure G(T, P) was determined by *G* (*T*, *P*) =  *min_V_F*(*V*,*T*). Here, the notation signifies the search for a unique minimum value by varying the volume while keeping the temperature and pressure constant. In the context of the QHA, the Phonopy package was utilized to generate displaced supercell lattices for DFT calculations and to estimate force constants. Using the relaxed atomic fractional positions, the relaxed volumes were varied systematically, with scaling factor 6 × 10^−6^ at 300 K. The equilibrium volume, denoted as *V*, at a given temperature *T* was determined when the Gibbs free energy G(T, P) reaches its minimum value.

## Conflict of Interest

The authors declare no conflict of interest.

## Author Contributions

K.G. and J.Z. contributed equally to this work. K.G. and H.Z. conceived and designed the experiments. J.Z., R.L., and H.Z. carried out the theoretical calculations and the data analysis. X.Y., Y.J., and X.Y. conducted the sample synthesis and data collection. J.Z., X.Y., Y.J., Y.L., and Y.Z. analyzed the data and drew the figures. K.G. drafted the manuscript and acquired financial support. S.L., J.L., W.L., and H.Z. polished the manuscript. All the authors have discussed and agreed upon the presentation of the manuscript.

## Supporting information

Supporting Information

## Data Availability

The data that support the findings of this study are available from the corresponding author upon reasonable request.
