# Supplementary material for: In‐Plane Overdamping and Out‐Plane Localized Vibration Contribute to Ultralow Lattice Thermal Conductivity of Zintl Phase KCdSb
Source: Adv Sci (Weinh). 2024 Jul 1;11(33):2402209. doi: 10.1002/advs.202402209 (PMC11633356; doi:10.1002/advs.202402209)
Supplement: Supplementary file 1 — Supporting Information [file ADVS-11-2402209-s001.docx]

Supporting Information

In-Plane Overdamping and Out-Plane Localized Vibration Contribute to Ultralow Lattice Thermal Conductivity of Zintl Phase KCdSb

Kai Guo*, Juan Zhang, Xiaotong Yu, Yuanxin Jiang, Yang Li, Yuqi Zeng, Ruixiao Lian, Xinxin Yang, Shuankui Li, Jun Luo, Wen Li, Hao Zhang*

**
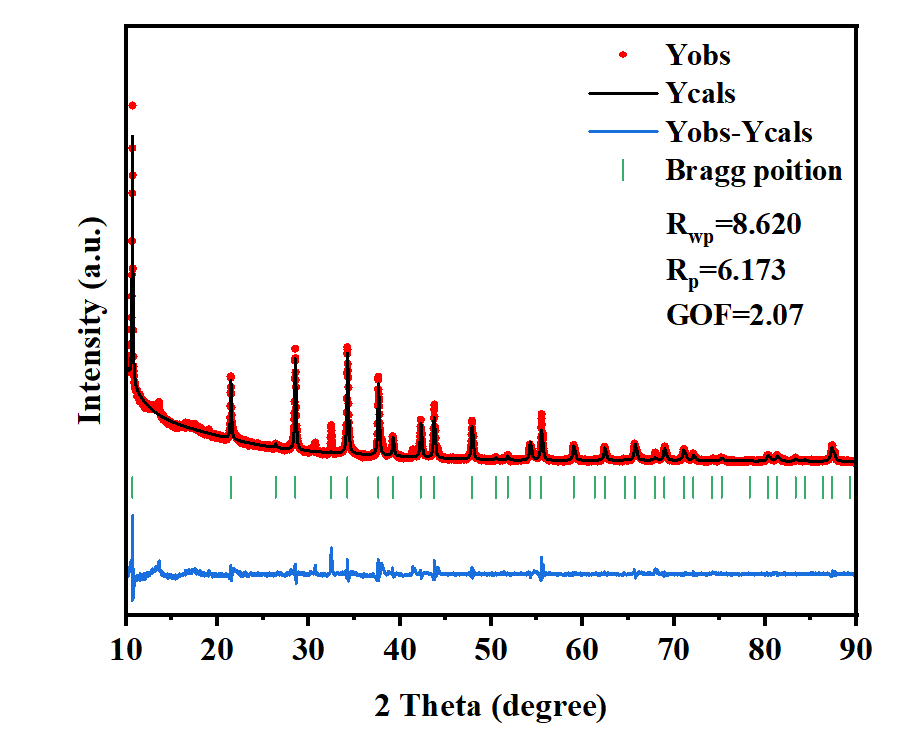
**

**Figure S1**. Structure refinement results of KCdSb.

The as-synthesized sample KCdSb has a tetragonal crystal structure with the space group of P4/nmm, since all major diffraction peaks in the XRD patterns are well-indexed, with the exception of a weak peak around 30 degrees, which is likely attributed to KOH from hydrolysis. The lattice parameters are calculated as a = 4.7777(1) Å, c = 8.2773(3) Å based on the Rietveld refinements. Weak preferred orientation is identified in the sample.


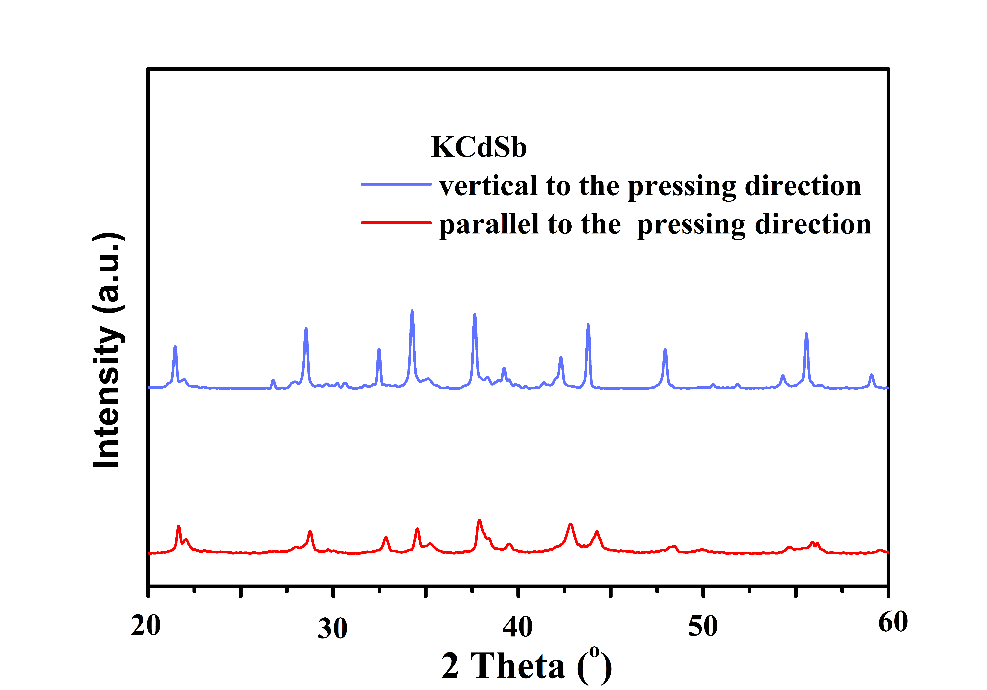


**Figure S2**. The XRD patterns of KCdSb polycrystalline samples measured perpendicular and parallel to the pressing directions.


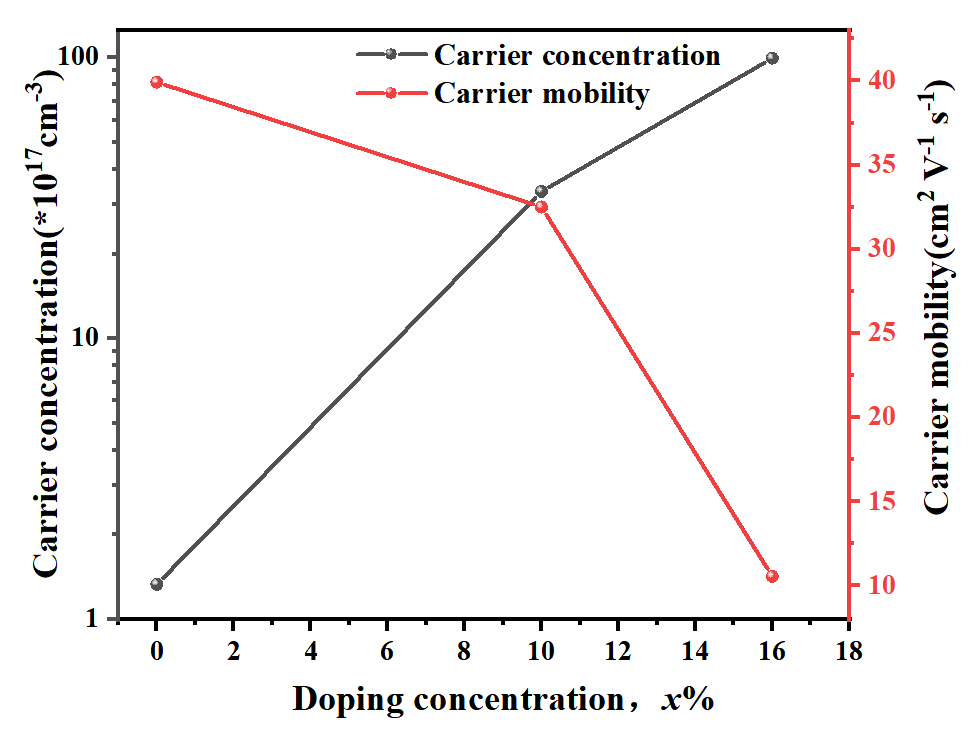


**Figure S3**. The Hall carrier concentration and mobility of Na alloyed KCdSb at room temperature.


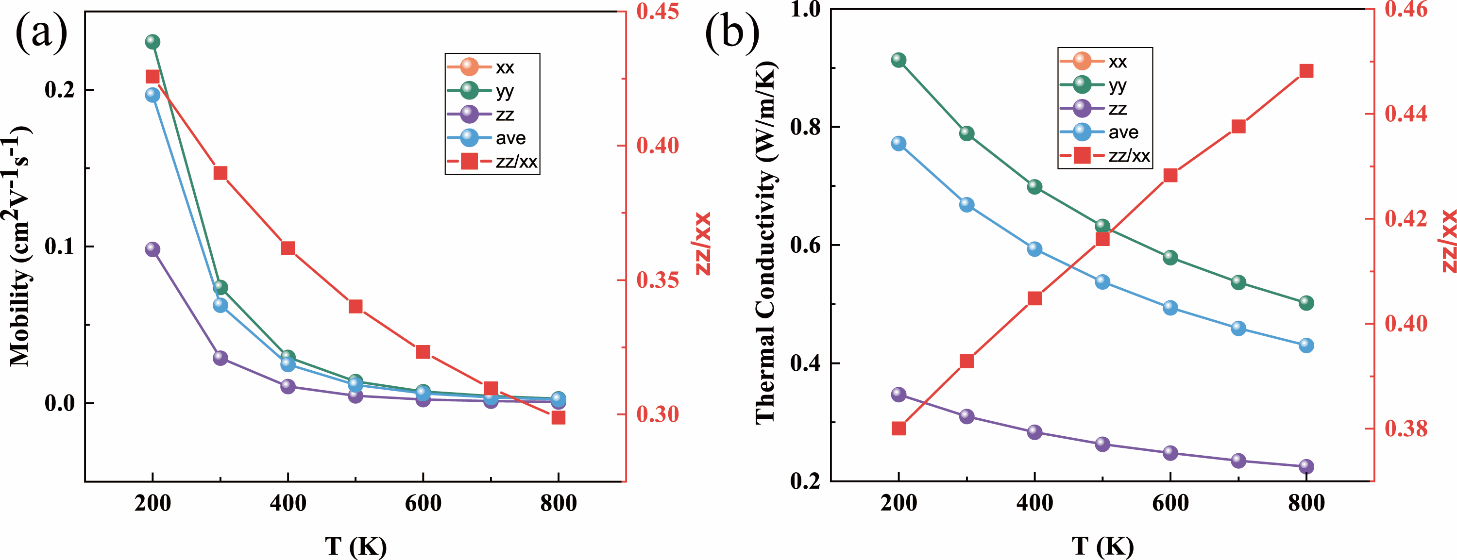


**Figure S4**. The anisotropic transport properties of KCdSb achieved by ab-initio calculations.
